# Adult Cleaner Wrasse Outperform Capuchin Monkeys, Chimpanzees and Orang-utans in a Complex Foraging Task Derived from Cleaner – Client Reef Fish Cooperation

**DOI:** 10.1371/journal.pone.0049068

**Published:** 2012-11-21

**Authors:** Lucie H. Salwiczek, Laurent Prétôt, Lanila Demarta, Darby Proctor, Jennifer Essler, Ana I. Pinto, Sharon Wismer, Tara Stoinski, Sarah F. Brosnan, Redouan Bshary

**Affiliations:** 1 Max Planck Institute for Ornithology, Department of Neurobiology, Seewiesen, Germany; 2 Department of Biology, University of Neuchâtel, Neuchâtel, Switzerland; 3 Department of Psychology and Language Research Center, Georgia State University, Atlanta, Georgia, United States of America; 4 Zoo Atlanta, Atlanta, Georgia, United States of America; 5 Institute of Evolutionary Biology and Environmental Studies, University of Zurich, Zurich, Switzerland; CNR, Italy

## Abstract

The insight that animals' cognitive abilities are linked to their evolutionary history, and hence their ecology, provides the framework for the comparative approach. Despite primates renowned dietary complexity and social cognition, including cooperative abilities, we here demonstrate that cleaner wrasse outperform three primate species, capuchin monkeys, chimpanzees and orang-utans, in a foraging task involving a choice between two actions, both of which yield identical immediate rewards, but only one of which yields an additional delayed reward. The foraging task decisions involve partner choice in cleaners: they must service visiting client reef fish before resident clients to access both; otherwise the former switch to a different cleaner. Wild caught adult, but not juvenile, cleaners learned to solve the task quickly and relearned the task when it was reversed. The majority of primates failed to perform above chance after 100 trials, which is in sharp contrast to previous studies showing that primates easily learn to choose an action that yields immediate double rewards compared to an alternative action. In conclusion, the adult cleaners' ability to choose a superior action with initially neutral consequences is likely due to repeated exposure in nature, which leads to specific learned optimal foraging decision rules.

## Introduction

The ecological approach to cognition proposes that a species' ability to solve a particular problem is tightly linked to its evolutionary history and, hence, to the ecological conditions under which it was selected [1]–[3]. A classic example is the tight link between spatial memory abilities and the dependency on food caching in corvids [4]. The ecological approach provides a general functional theoretical framework which allows for the integration of studies on any animal species, including invertebrates, such as the demonstration of sophisticated spatial orientation skills of bees [5], and the ability of jumping spiders to plan where to go to in order to attack prey [6]. The ecological approach has led to a great diversification of animals studied, and in particular to the appreciation that animal clades that lack particularly large and complexly structured brains may provide examples of impressive cognitive abilities. This is in particular true for fishes [7], which have provided some excellent examples for complex social strategies. Male cichlids (*Astatotilapia burtoni*) use transitive inference to predict fighting abilities of competitors [8] and sticklebacks (*Pungitius pungitius*) employ so-called hill climbing social learning strategies [9], in which they compare their own foraging success with the success of observed individuals to update foraging decisions. Another example involves the foraging decisions of cleaner wrasse, *Labroides dimidiatus*. These cleaner fish occupy small territories (so-called ‘cleaning stations’) in which they interact with a variety of reef fish species (so-called ‘clients’) from which they remove ectoparasites, but also mucus and scales [10]. Conflict occurs because cleaners prefer to eat mucus over ectoparasites [11], where eating the former constitutes cheating (for a review of cleaners' decision rules, see [12], [13]). Cleaners adjust levels of cooperation to the strategic options available to clients to react to cheating by cleaners. Predatory clients typically receive the highest service quality, whereas non-predatory resident clients, who lack choice options, punish cleaners for cheating. Visiting clients who have access to alternative cleaning stations receive faster service than resident clients that have access to only one cleaning station. This is because visiting clients represent an ephemeral food source: they may swim off and visit another cleaner for their next inspection if not inspected immediately. In contrast, resident clients must wait for inspection because of a lack of alternatives. Furthermore, cleaners pay attention to the presence of potential clients and are more cooperative to current clients if that allows them to access bystanders [14]. Thus, cleaner wrasse show high adaptation to the specifics of an interaction in their foraging decisions, which are at the same time linked to interspecific social behavior. The precision with which cleaners adapt current service quality to current conditions may be predicted by their ecology: cleaners have over 2000 interactions per day with a great variety of clients and fully depend on cleaning for their diet [15], thus their performance during the interactions has a major impact on their fitness. However, the ecological approach is rather nonspecific with respect to the cognitive processes that underlie the performance. Hence, we cannot infer from the precision and flexibility in cleaner foraging decisions that they warrant much learning, memory or comprehension and hence, ultimately any adaptive changes in corresponding brain areas. In addition, we do not know whether reaching their food maximizing decisions involves widespread learning rules or whether rather specific abilities must be evolved or developed. Thus the question of interest is whether any (vertebrate) species could easily behave like a cleaner wrasse if it switched its diet to ectoparasites and mucus of fishes, or whether specific selection pressures on cleaner wrasses have caused specific abilities? And if specific abilities do exist in cleaner wrasses, what is the role of cognition?

Here, we provide the first test of the hypothesis that cleaner wrasse foraging decisions are the result of specific cognitive abilities. Our laboratory experiment involved two identical food sources – two plates differing in colour and patterns to allow discrimination, but providing exactly the same food - where one source (plate) was ephemeral and the other one permanent. This mimicked the simultaneous visit of a resident and a visitor to the cleaning station. Accordingly, the food maximizing solution involved eating from the ephemeral food source first and only then from the permanent one. The potential difficulty of the task is due to the fact that no matter which plate an individual chooses first, it will receive exactly the same immediate reward, and only then will it (possibly) have the chance to perform a second act that would yield an additional reward. Thus, the initial decision may not lead to reinforcement learning unless an animal is somehow able to integrate the future consequences into its immediate decision. Despite theoretical considerations indicating that the task is not trivial to solve, a previous study suggested that cleaners could quickly solve it, though individual learning was not investigated [16]. In order to test whether the ability to solve the task is linked to its ecological relevance and whether the solution by cleaners reflect specific learning rules, we subjected both adult and juvenile wild-caught cleaners as well as three primate species to the task. The comparison between adult and juvenile cleaners allowed us to address the potential role of individual experience. Client composition shifts during ontogeny, with adult cleaners interacting about three times more frequently with visitors than do juveniles (comparing data published in [17], [18]). Thus, juveniles rarely experience the situation in which a visitor and a resident seek cleaning simultaneously. Therefore, if adult cleaners perform better than juveniles that would indicate that individual experience in the field helps to solve the abstract laboratory task.

An important aspect of the ecological approach is to test whether other species that do not engage in cleaning interactions are less able to solve the task. We decided to use primates – capuchin monkeys, chimpanzees and orang-utans – for the comparison for several reasons. First, the general circumstances of the cleaners' decisions involve social interactions and foraging, which matches the two contexts that have been proposed to select for large brains in primates [19]–[22]. Second, primates, and in particular our three study species, have been shown to possess a large array of cognitive mechanisms in the context of social behavior and foraging. Specifically, all three species have a complex diet and have been classified as extractive foragers [21]. In addition, at least chimpanzees and capuchins hunt for meat and catch mobile insects and reptiles [23]–[25], and in doing so, encounter ephemeral food sources. Moreover, all three species are able to solve some cooperation tasks in the laboratory [26]–[32], and capuchins and chimpanzees do so in the wild [23]–[25], [33]. Also, our task involved the ability to take not only immediate but also future consequences into consideration, an ability that primates have repeatedly demonstrated in foraging experiments (delayed rewards experiments: [34], [35]; planning experiments: [36]–[39]). Finally, all three of our primate study species have large brains compared to other species, and large relative brain sizes (e.g., brain-to-body or neocortex-to-body ratios) even compared to other primates [40], again indicating high general cognitive abilities.

Although evidence suggests that the primates will excel in tasks that involve future consequences in the context of cooperation and foraging, the specifics of our task may favor cleaners. For example, cooperation and foraging are intertwined in cleaners in a way that is absent in primates; most importantly, cleaners cooperate with their food sources. In addition, primates encounter ephemeral food sources (e.g., insects, small vertebrates) unpredictably and opportunistically, and thus the ecological constraints are quite different from those of the fish, for whom the interaction with ephemeral sources is predictable. Based on this, we predicted that unlike the cleaner wrasse, the primates would not perceive the task as a social interaction but just as an optimal foraging task. Thus, our experiment offered us the opportunity to test the ecological intelligence hypothesis in a quite specific way. We expect that if ecology is the driving force that helps to solve the problem, then cleaners should individually learn to solve the tasks faster than any of the primate species. Conversely, if the general context and brain size (relative or absolute) prepare better for the task than rather specific ecological conditions do, then the primates should learn to solve the task faster than the cleaner wrasse. We also considered an additional way to test the role of learning for the cleaners' decision making process, reversing the role of the two plates once an individual reached the learning criterion. The former permanent plate now became the ephemeral plate and vice versa. Although cleaners are able to discriminate between different client categories, including resident and visitor, and can even individually recognize clients [13], reversal of roles does not occur under natural conditions, i.e. a visitor individual/species never turns into a resident. Therefore, it appears to be highly unlikely that reversal learning could be aided by the adaptation of an innate program. For the primates we included this task only to see whether once the task has been solved, they understood its general principle. We predicted that if primates found the task initially difficult but solving it triggered a more general understanding, then their performance would greatly improve during the reversal.

## Results

### Initial learning tests

**Figure 1:**
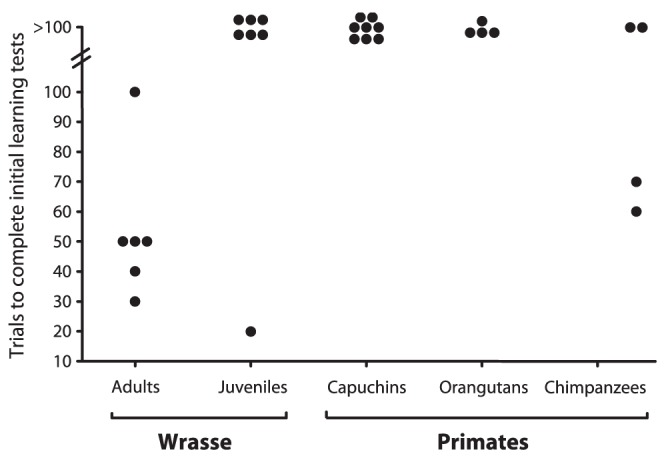
The number of trials required for individuals to learn to eat first from the ephemeral plate. Dots represent an individual. The y-axis indicates the number of trials required to learn the task.

All six adult cleaner fish individuals learned to eat first from the ephemeral plate, which was smoothly withdrawn if the cleaner were to forage on the permanent plate first. Individuals took 3–10 sessions (of 10 trials each) to reach the criterion of significance with a median of 4.5 sessions. In contrast to the adult cleaners, only one juvenile cleaner and two out of four chimpanzees solved the task within 10 sessions, and all other subjects failed (Fig. 1). Thus, there was a significant difference in learning speed between the species/age classes (Kruskal-Wallis Test: df = 4, H = 18.4, p = 0.001). Post-hoc comparisons revealed that adult cleaners performed better than juvenile cleaners or any of the three primate species (Student Newman-Keuls, all p<0.05). Most of the primates that failed to learn the task developed a strong side preference (7/8 capuchin monkeys, 3/4 orang-utans and 1/2 chimpanzees). Juvenile cleaners that failed the task developed a preference for the permanent plate. All primate subjects that had failed to learn the task within 10 sessions (100 trials) were then exposed to changes in the experimental design to learn the solution. The details varied between species and are described in Information S1. Under the altered conditions, all capuchin monkeys and three out of four orang-utans eventually developed a significant preference for the ephemeral plate while the two remaining chimpanzees failed to learn the task at all. We then included the capuchins and the three orang-utans in the reversal learning task.

### Reversal learning

For this component of the experiment, the previously ephemeral plate/tray became the permanent plate/tray, and vice versa. All the adult fish developed a significant preference for the new ephemeral plate within 10 sessions (median: 7; ranging: 6–9; Fig. 2). With one exception it took individuals slightly longer to re-learn the task after the plates suddenly inversed their behavior (reversal learning phase) as compared to learning the initial behavior of the plates (exact Wilcoxon signed rank test, n = 6, W = −13, p>0.05). The one juvenile that succeeded in the initial task after only 20 trials apparently had had a preference for the initial ephemeral plate: it failed to alter its preference over the next 100 reversal trials. Seven out of eight capuchins learned the reversal task in 6–9 sessions, yielding similar results to the adult cleaners. In contrast only one orang-utan out of three and neither of the two chimpanzees learned the reversal task within 10 sessions. Overall, there was a non-significant difference in learning speed between the species (Kruskal-Wallis Test excluding the one juvenile cleaner, df = 3, H = 6.8, p = 0.078). If the few chimpanzee and orang-utan individuals were pooled as ‘apes’ the differences between species became significant (Kruskal-Wallis Test excluding the one juvenile cleaner, df = 2, H = 6.5, p = 0.038). Post-hoc comparisons revealed that both adult cleaners and capuchin monkeys performed significantly better than the apes (Student Newman-Keuls, both p<0.05). Both chimpanzees that failed the test developed a significant side bias, whereas the orang-utans did not develop a discernable bias. The apes' unexpected lack of success appeared to be due to frustration with the task [41], [42].

**Figure 2:**
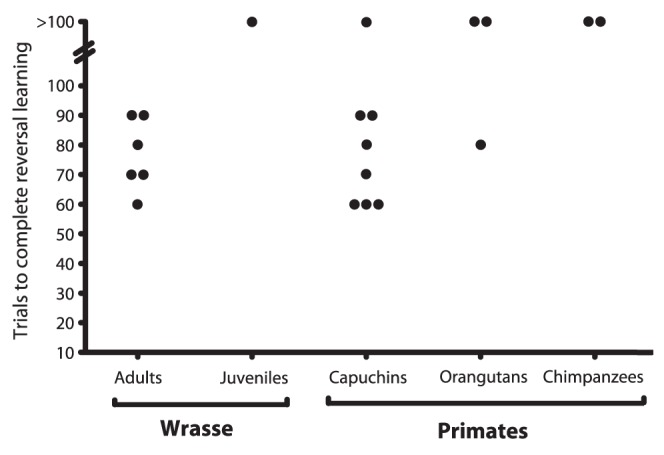
The number of trials required for individuals to reverse their preference when the plates switched roles (e.g., the permanent tray became ephemeral and the reverse). Again, dots represent an individual, and the y-axis indicates the number of trials required to reverse the preference.

## Discussion

A key conclusion from our experiment is that the sophisticated foraging decisions which cleaner wrasses demonstrate during interactions with client reef fish are not easily achieved by other species with larger and more complexly organized brains. The ability to choose between an ephemeral and a more permanent food source of otherwise identical quality is apparently far from simple as the vast majority of individuals from three primate species that otherwise excel in cognitive tasks failed to learn the task within 100 trials, as did juvenile cleaners. However adult cleaners consistently solved the task. Thus, our task differs from experiments that demonstrate extremely fast learning of solutions if individuals are placed into a key stimulus-response context, in which even invertebrates like bees may outperform primates, including humans [42]–[43].

### Why the task may be difficult to solve

When confronted with a choice that directly yields two different amounts of food primates can easily discriminate outcomes with one reward from those with two [44]–[47] (for that matter, fish can do the same [48], [49]), even in cases in which the quantity to be received is indicated symbolically (e.g., via tokens or Arabic numerals [50]–[52]). Thus there must be another explanation for the decrement in performance in the primates as compared to the adult wrasses. We consider several possibilities for why this task may be difficult to learn. First, assuming that both species saw the task as a sequence of two tasks (rather than one task with two steps, a reasonable assumption since they got fed after their first choice, and hence before their second), then the difficulty of the task may relate to known reinforcement mechanisms; in this case, no matter which plate an individual chooses first, it will receive exactly the same immediate reward, and only then will it (possibly) have the chance to perform a second act that would yield an additional reward. That is, our design, compared to classic associative learning designs (i.e. go to A, then to B, then collect reward), adds the complication of requiring animals to go to B to collect a second reward after A already has been rewarded. Thus it is possible that the first plate chosen becomes a conditioned stimulus that is stronger, as it is always the first stimulus to be rewarded (and thus may result in the greatest satisfaction). After this, there may be little novelty or information value left for the second plate, lowering the incentive as compared to the first plate/reward. Thus phenomena like blocking (e.g., little conditioning is occurring) or overshadowing (e.g., less conditioning is occurring to this weaker conditional stimulus) might explain why there seem to occur little learning about the second plate if the first plate already has been rewarded.

Second, it is possible that the fish experienced the removal of the plate as a stronger punishment than did the primates. Both the fish and the primates presumably reacted to the removal of the second plate, containing food, as a negative reinforcer (e.g., punishment). However, fish may have additionally experienced it as a social punishment; one indication that they indeed perceive the task as a cleaning situation is that they respond with tactile stimulation when the plate returns, a behaviour cleaners use to reconcile and to make clients stay longer under natural conditions [53] to encourage it to stay this time. Hence negative social reinforcement (or: social punishment) would make the task more aversive, and hence easier to learn, for the adult fish as compared to the primates and juvenile fish, both of which have far less experience with this situation.

Finally, a more cognitive mechanism than associative learning that would allow subjects to solve the task is insight based on backwards induction. In backwards induction, one has to start with the desired endpoint and then figure out which steps lead to that endpoint. Evidence for backwards induction has been demonstrated in a chimpanzee, Julia, who had to open up to 10 Plexiglas boxes with specific tools inside in the right sequence to finally obtain food in the last box [36]. However, the primates in our study apparently failed to use backward induction, despite a large number of trials. Given the evidence for insight learning in our primate species, why did they fail to use this ability? One possibility again relates to reinforcement; Julia was not rewarded for each step of her process, while in our experiment the subjects were. As discussed above, it is possible that the receipt of intermediate rewards interferes with learning mechanisms in that it lowers the incentive value of the second reward [54].

We finally note that the apes' unexpectedly low performance on the reversal task was likely due to frustration with the procedure. Apes – including some of these subjects – are typically very good at reversal learning tasks [41], [42]. Moreover, within the primates, reversal learning performance is associated with brain size [55], and apes typically outperform capuchins [56]. However, our subjective impressions indicated that the apes found this task very frustrating. Despite there being only 10 trials in a session, we initially had to change the ITI from 5 minutes to 90 seconds in order to get them to complete a session (see Methodological considerations, below, for a more detailed discussion of this). Even with the 90 second ITI, by the later sessions, apes were hitting or grabbing the choice trays rather than choosing a reward, and often refused to participate. We believe it was this frustration with the task that caused the unexpectedly low performance on reversal learning. What is perhaps more notable is that the fish did so well. Their behavior is counter to that predicted by the primates' association between reversal learning and brain size [55] and deserves far more attention as a potential area in which fish cognition equals that of the larger-brained primates (see Methodological considerations, below, for other areas in which fish cognition appears to equal that of far larger brained species).

### Why adult cleaner wrasses may have been able to solve the task

We propose two non-mutually exclusive explanations for why adult cleaners learned to solve the task. First, the cleaners may have developed the decision rule to preferentially approach ephemeral food under natural conditions and then applied the same rule to this task. In contrast, the primates were born in captivity, where sufficient food is provided multiple times per day (at all facilities) and they rarely catch ephemeral food like invertebrates. Second, as discussed above, the cleaners may have perceived the task as a social interaction. In that case they would have perceived the removal of the ephemeral plate as the loss of a cooperation partner and hence as a negative reinforcer that reduced the likelihood that the subject would choose the permanent plate again on future trials. The aversion to losing any client would make the ephemeral plate more attractive to cleaners, whereas primates are not selected to experience either the negative reinforcement of a missed opportunity or social reinforcement for interacting with their foraging substrate. Thus, we consider it likely that cleaners, but not the primates, simultaneously experienced a positive and a negative reinforcer, which would explain why they learned to solve the task rather quickly as compared to the primates. If that was the case, a change in protocol for the primates that let them perceive the interaction as social (for example by replacing the trays with human partner) should yield much faster learning.

If our hypotheses are correct then one would also predict that even individuals of the closely related cleaner wrasse species *L. bicolor* should have problems solving the task. This is because adult bicolor individuals rove over large areas and typically approach the clients they want to interact with rather than having to wait for them at a cleaning station [57]. Thus, the distinction between residents and visitors is not crucial to them, and they can follow clients that are about to leave in order to prolong interactions. For bicolor individuals, it appears to be mainly important where an interaction takes place within their home range: they are more cooperative in their core area than in the periphery [58]. To explore this hypothesis, we additionally collected preliminary data on *L. bicolor*. We tested two individuals in May 2009 at the University of Neuchâtel following exactly the same protocol as we used for adult *L. dimidiatus*. One bicolor failed to learn the initial task within 200 trials. The other one learned the initial task in 70 trials but failed at the reversal: after a short period of random choices it redeveloped a preference for the initially ephemeral plate. Taken together, there is thus a significant difference in overall performance (in trials to complete the entire experiment) between the two species (Mann-Whitney-U-Test, m = 6, n = 2, U = 0, p<0.05). Clearly, more bicolor individuals should be tested (unfortunately, they are very difficult to obtain from licensed commercial pet shops; three individuals were all we managed to obtain over a six week search period, with one not willing to participate in the experiment). Nevertheless, the preliminary results suggest that the ability of *L. dimidiatus* individuals to solve the task is linked to very specific ecological conditions that are not met in *L. bicolor*.

### A comparison between juvenile and adult cleaners

There are various potential explanations for why juveniles failed to solve the task while adults managed. One possibility is that maturation processes in the brain preclude juveniles from solving the problem at hand. Second, there were small differences in the experimental protocol due to different research sites and in turn testing possibilities: juveniles – kept on Lizard Island for the period of the experiment - experienced longer time intervals between subsequent trials as compared to the adults which where housed and tested in Neuchâtel, Switzerland. However, in an earlier experiment adult cleaner fish that were trained on a similar task (i.e. “one plate remains until inspected while the other does not”, p. 132), but with 30 min intervals between trials, significantly chose to first clean the plate that would not wait until being inspected [16]. Thus we doubt that the differences in the ITI are reason enough for the differences in learning performance.

While maturation and (to a lesser extent if at all) experimental design may have affected the results, we consider it likely that individual experience plays a major role; juveniles have fewer visiting clients and are therefore rarely in a situation that calls for this discrimination. The situation changes for adults; in a field study, adults had to make choices between a visitor and a resident client more than twice per hour (120 times in 52 hours of observation [13]; our subjects were wild caught). It has long been known that maturation and experience combine to determine performance [59]. But only recently has it been shown that, for example, guppies possess from birth on numerical abilities (discrimination of small numbers), which unfolds as a result of both maturation and social learning (discrimination of larger numbers) [60]. A logical follow up experiment should therefore test adult cleaners that have been kept in captivity without simultaneous exposure to residents and visitors.

### Methodological considerations

We note that the primates consistently performed poorly despite the fact that we ultimately adapted the methods to be as appropriate as possible for each species (within the constraints of using trays with different ‘behaviors’ to present identical foods). In particular, the capuchin monkeys received several different methodologies as we attempted to optimize a protocol which allowed them to eventually solve the initial task. Variables that seemed to have helped them included a barrier between the plates and much shorter time intervals between trials (Table 1). These shorter inter-trial intervals may have reduced the cost of an incorrect choice for the primates, potentially making it less likely for them to learn the task. However, we note several things which argue against this possibility. First, in the initial phase of pilot testing with capuchin monkeys, all subjects received ten sessions of ten trials each (100 total trials, a trial number which allowed all adult cleaners to learn the task) with 15 minute ITIs between each trial. No subjects' choices differed from chance (binomial test, all ps<0.05 both for individual sessions and when sessions are combined). Four additional subjects received an additional four session (40 total trials, for a cumulative total of 140 trials) with 15 minute ITIs between each trial, and again, no subject's choices differed from chance (all ps<0.05). Finally, in the last phase of testing, in which subjects learned the test, ITIs were reduced to 5 minutes (still with 10 trials per session, so an additional 100 trials total). We note that there were several other factors that changed between these tests. First, of course, there is an experience effect; however even with 100 trials with 15 minute, no capuchin learned within 100 trials, yet all of the fish did so. Additionally, in the first phase only, the ephemeral tray was pulled back, but not removed from sight as it was in later phases. While this may have confused the primates, they are accustomed to food rewards being visible, but unavailable from their daily life. In fact, being able to see the food that they could not access could arguably have increased the magnitude of the negative reinforcement for choosing the other plate, possibly supporting learning. Finally, there was no divider between the trays in the first phase of testing, which may have made it more difficult for the primates to discriminate between the choices. However, subjects had to reach through one of two discrete doors, actively pushing open the door in the process, so it is difficult to see how they could not discriminate between the options.

**Table 1:** A summary of information about subjects and experimental protocol.

	Adult wrasse	Juv. wrasse	Chimpanzees	Orang-utans	Capuchins
**General**					
N individuals	6	7	4	4	8
Date	3–4/09	7–8/10	8/10–4/11	8/10–4/11	8–12/09
Location	Neuchâtel, CH	Lizard Island, AUS	LRC GSU, USA	Zoo Atlanta, USA	LRC GSU, USA
**Experiments**					
Time between trials	15 min	30 min	90 sec	1. 90 sec	1. 15 min
				2. 30 sec	2. 5 min
Subject order	varied	varied	varied	varied	varied
Plate color	red-yellow	green-grey	blue	blue	green-blue violet-yellow blue-yellow
	green-white	pink-grey	yellow	yellow	
Plate side counterbalanced	yes	yes	yes	yes	1. no
					2.yes
Food type	mashed prawn	mashed prawn	banana	cheerios cereal	apple
Plate preference test	yes	no	yes	yes	no
Food already on plate/tray	yes	yes	yes	no	yes
Removed plate/tray	out of view	out of view	out of view	out of view	1. visible
					2.out of view
**Initial learning test**					
Maximum N sessions	10	10	10	10	10
Trials per session	10	10	10	10	10
N sessions per day	2	2	1	1	1
N test days per week	7	7	3	5	3
**Reverse learning test**					
Maximum N sessions	10	-	10	10	10
Trials per session	10	-	10	10	10
**modifications**					
				reload ephemeral tray 10× per trial	Counterbalance, tray shape/color
				shorter time intervals	
			Cardboard barrier between plates		

The capuchins were all tested prior to the tests with the apes, and as much as possible we used the final capuchin protocol for both ape species. The choice tray featured a divider, and non-chosen options were immediately removed from sight. One thing that we could not do similarly was the five minute ITI. In pilot testing, the chimpanzees and orang-utans reacted with extreme frustration to a 5 minute delay, leaving the testing area and refusing to return. Thus we shortened the ITI to 90 seconds to encourage subjects to participate. If, as we found, the capuchins' behaviour was positively influenced by the shorter ITI, then a shorter one yet for the apes should have made the task easier. Additionally, while this might have resulted in less cost per choice, subjects still only receive 10 trials per session, so there were very few chances to receive treats during testing (in most cognitive tests, subjects receive at least two to three times this many trials in a session). Overall, this meant that the details of the procedure were optimized during the course of the study for the primates, but not the fish. Therefore, we consider it unlikely that cleaners outperformed primates due to advantages with respect to methodological details like the color or shape of trays, the food, or the inter-trial interval.

Finally, note that the primates acquired food by reaching out and grasping it, while fish swam to different foods and took them directly into their mouths. This was due to differences in body plan between fish and primates. Fish have to move between compartments with their whole body, but from where they were located could easily see both rewards simultaneously. Due to the size of the primates and caging constraints, it was impossible to house them such that they could simultaneously see both rewards and be housed in a third room. This would be particularly problematic for our study if they could not immediately view the ephemeral reward being removed when they chose the permanent reward first. Moreover, this procedure would have meant that the primates had far longer time intervals to both access the first reward and between the first and second. Additionally, primates typically make choices by grasping with their hands. Of course, when comparing species with very different body plans and abilities, identical procedures may be difficult or impossible, both for practical reasons (e.g., the presence or absence of hands) and differences in experience or ways of interacting with the world. In particular in cases such as ours, in which a species performs differently than expected, we encourage the use of multiple procedures in an effort to optimize the design for the species, even if this results in some methodological differences.

In conclusion, our results provide the first evidence that cleaners' sophisticated behavior in cleaning interactions is due to selection for specific rule learning that require experience and/or maturation. All three primate species have a complex diet and are known to cooperate, but still they were outclassed by adult cleaners in this foraging task. Although we cannot entirely rule out differences in procedure that resulted from the comparison between very different species, a possible mechanism underlying the fishes' response is that they perceive the leaving of a food source as a negative reinforcer, and therefore choose the ephemeral food source first before approaching the permanent one. This implies that the specificity of the cleaners' ability to give priority to ephemeral food sources lies not in a sophisticated cognitive process but in the ability to identify relevant stimuli. Nevertheless, recent research on fishes has yielded evidence for various supposedly more complex cognitive abilities (reviewed by [61]). As mentioned before, nine-spined sticklebacks use social learning rules that compare own success relative to the success of potential models [62], [63], and male cichlids may use transitive inference to assess the strength of potential rivals [64]. But there are many other examples. Various coral reef fishes have spawning traditions [65], [66]. Fathead minnows show the ability to generalise between predators [67]. Groupers signal their intention to hunt to moray eels in the absence of prey [68]. Guppies' performance in relative quantity judgments adheres to that of humans tested in non verbal numerical tasks [69], and also mosquitofish can use numbers like primates [70]. On the neurophysiological level there is recent evidence that the reward structure of fish brains is similar to that of mammals: hedonistic rewards like receiving tactile stimulation may yield fitness advantages [71]. With respect to cleaner wrasse, we note that they express many abilities in the context of cleaning interactions, including adjustment of service quality to the presence of a co-inspecting partner [72] and to the presence of an audience [14], the use of predatory clients as social tools against chasing non predatory clients [73] and the ability to remember the ‘when’ and ‘what’ of interactions [74]. These phenomena are not specific to cleaner wrasse – client interactions, and they have been linked to more complex cognitive processes like social awareness [75] and an understanding of other individuals as agents [76] in studies on primates. It will therefore be of interest to use cleaner wrasse to test in how far such higher cognitive processes might be present or absent in a ‘lower’ vertebrate that is nevertheless under similar selective pressures of a complex social environment.

## Methods

Experiments on adult cleaner wrasse were carried out in March and April 2009 at the University of Neuchâtel, Switzerland, while juvenile cleaner wrasse were tested at Lizard Island Research Station, Australia, in July and August 2010. Experiments on capuchins (August to December 2009) and chimpanzees (August to December 2010) were carried out at the Language Research Center, Georgia State University, USA, and orang-utans were tested at Zoo Atlanta, USA (August to December 2010).

### Subjects and housing conditions

#### Cleaner wrasse

Six adult wild caught *Labroides dimidiatus* (5.5–7.6 cm total length; TL) of unknown sex were purchased from a licensed pet shop. All adults were individually housed in aquaria measuring 60×30×30 cm^3^ in size and filled with approximately 25 l of saltwater (details regarding water available in Information S1). Juvenile cleaners (1.5–4.5 cm; TL) were caught with hand-nets from reefs surrounding Lizard Island and housed individually in glass aquaria (62×27×37 cm^3^) with a continuous flow of fresh sea water. All cleaners were supplied with an opaque Plexiglas shelter tube (length: 10 cm, Ø = 2.5 cm) for hiding during the day and sleeping at night. Cleaners were first trained to feed off grey Plexiglas plates, and fed *ad libitum* every day to ensure sufficient food intake independent of their performance during experiments. All individuals were initially fed mashed prawn flesh or a mixture of mashed prawn flesh and fish flakes. Individuals were kept for 10 days prior to commencing experiments.

#### Primates

All primates were captive born. The eight brown adult capuchin monkeys (5 males, 3 females, age range 5–20 years, median age of 10 years) were from two separately housed social groups at the Language Research Center of Georgia State University, USA. The four chimpanzees (2 males, 2 females, age range 25–40 years) were also from the Language Research Center, whereas the four orang-utans (3 males, 1 female, age range 7–33 years) were from Zoo Atlanta, USA. All primates lived in stable social groups consisting of adult male(s) and female(s) and any attendant offspring. They were separated from these groups only for behavioral and cognitive testing. Details regarding housing conditions are provided in Information S1. Subjects were fed a diet according to their species-specific needs, but generally consisting of primate chow and fresh fruits and vegetables. They also received enrichment-foods several times per day; consequently, animals were never food or water deprived for testing purposes. Running water was available *ad libitum* at all times. Subjects could choose not to participate at any time by walking away from the experimenter.

### General procedure

The experimental design was based on a study by Bshary & Grutter [16]. Some variation occurred in the way the three primate species were tested (Table 1). Both fish and monkeys had to make an initial choice between two visually distinct food plates/trays, both offering the same food in equal quantity: 0.0001–0.001 g pieces of prawn (highly preferred food) for the fish, 0.5×0.5×0.5 cm^3^ dried apple pieces for the capuchins, cheerios for the orang-utans and 5 mm slices of banana for the chimpanzees. Plates and trays varied in size, shape and color between species in species-appropriate ways (see Table 1). All plates and trays were attached to handles so that they could be moved towards subjects but also be retracted rapidly. The “ephemeral” plate would be removed immediately if not chosen first by the subject, whereas the “permanent” tray would remain until the subject had taken the food item. Which plate was which was counterbalanced between individual subjects within a species. The ephemeral plate mimicked visiting clients with access to several cleaning stations, which under natural conditions would leave if they were not inspected immediately [42]. Alternatively, the permanent plate mimicked resident client species that would line up to be cleaned [18]. Subjects readily interacted with both respective plates.

All cleaner wrasse were tested in their aquarium. A separation with a central sliding door was introduced at approximately two-thirds of the aquarium length to create an ‘experimental’ compartment and a ‘resting’ compartment. For cleaners, a given trial started by confining the subject to the smaller ‘resting’ compartment of the aquarium. After approximately 60 s, the client plates were placed at equal heights at the opposite end of the aquarium, i.e. the experimental compartment. After about 10 s, the door was opened and the cleaner could enter the experimental compartment at will.

For capuchin monkeys, members of each social group, consisting of four subjects, were simultaneously tested in separated test chambers attached to their home enclosure. Monkeys had previously been trained to be separated from their social group and to individually enter these chambers, where virtually all testing was done. Dependent offspring were always allowed into the testing area with their mothers. Testing chambers measured 61×44.5×33 cm^3^ in size and were separated from each other by approximately 40 cm. The test chamber was backed by an opaque panel, allowing vocal, but no visual or tactile, access to their group. This allowed us to interact with subjects in a controlled manner with minimal distractions from the group. The sessions for the apes were organized in a similar way: subjects were all tested in a subsection of the indoor section of their home enclosures, while still in auditory and visible contact with the other group members (this is how all testing is done at these facilities). The order in which subjects were tested varied from day to day. As with the capuchins, dependent offspring were always allowed into the testing area with their mothers. Note that for all species, acquiring the food required accessing a separate area from where the subjects were initially located. For fish, this required swimming, while for the primates this required reaching outside of the compartment.

### Presentation of plates

The position of the two plates was randomized, but with an equal number of presentations on each side within each 10 trial sessions. Randomization was constrained such that the same tray was never presented more than three consecutive times on the same side. (Note that capuchins were initially tested with the plates altering sides between sessions; see Table 1). The two plates were placed far enough apart that, following a choice of the permanent plate, the experimenter could remove the ephemeral plate before the subject could take the food. It proved impossible to put the trays far enough apart to stop the capuchins from grabbing both food items simultaneously, so we added trapdoors to allow access to only one at a time. These consisted of two doors attached to each other by a string that worked in a drawbridge-like fashion, pulling one door closed when the other was pushed open. No special constructions were required for the great apes, as the mesh structure of the cage prevented them from quickly grabbing both items simultaneously. The procedures were identical for the reversal learning phase, except that the plates' behavior was reversed abruptly, i.e., the previous ephemeral plate now behaved like the permanent while the previous permanent plate became the ephemeral plate. There were differences between experimental groups concerning the number of sessions per day (one or two; all of ten trials), the number of testing days per week (every day for cleaners, but 5 days per week for orang-utans and 3 days per week for capuchins and chimpanzees), and the time interval between successive trials (15 or 30 min for the cleaners and generally shorter for the primates). See Table 1 for specific details.

### Learning criterion

We based our significance criterion on Sign-Tests-Table (two-tailed). Significance was reached when a subject made correct choices on ≥9/10 trials on one session or ≥8/10 on two or ≥7/10 trials on three consecutive sessions. For capuchins in the initial sessions, the criterion for learning was ≥16/20 trials (e.g., over 2 sessions) because plate positions were constant during a session and hence a side bias would have led to the inaccurate assessment of significant “learning” in half of sessions (and significant “anti-learning” in the others). Once an individual had reached criterion, we ran the reversal trials. We used the same criterion for the reversal test. We were primarily interested in relative performance rather than the question whether all subjects can learn to develop a food maximizing preference eventually if given sufficient opportunity. Adult cleaner wrasses were the first to be tested out of all the experimental groups. They formed the baseline for the others with respect to the questions we attempted to answer. As all of them learned to solve both the initial and reversal tasks within 100 trials, we fixed 100 trials as an upper limit for the other experimental groups. Because the reversal learning task required learning of the initial task, we decided to expose any primate that failed to learn the initial task within 100 trials to modified versions of the task. The modifications were designed to facilitate learning (see Table 1). Some of the modifications were included in the reversal learning task, such as re-baiting of the ephemeral plate for orang-utans when a subject chose correctly. In addition, as capuchins were the first primates tested, we adopted some of the experimental features that seemed to have helped them to learn the task (shorter between trial intervals, more visually distinct plates, counterbalanced presentation of plates within sessions) for the experiments on the great apes (see Table 1).

## Supporting Information

Information S1 (DOC)

## References

[1] Kamil AC (1998) On the proper definition of cognitive ethology. In Balda RP, Pepperberg I, Kamil AC, editors. Animal Cognition in Nature. San Diego: Academic Press. pp. 1–28.

[2] Bshary R, Salwiczek LH, Wickler W (2007) Social cognition in non-primates. In Dunbar RIM, Barrett LS, editors. Evolutionary Psychology. Oxford: Oxford University Press. pp. 83–101.

[3] Shettleworth SJ (2010) Cognition, Evolution, and Behavior, 2nd edition. Oxford: Oxford University Press. p. 720.

[4] Balda RP, Kamil AC (1989) A comparative study on cache recovery by three corvid species. Anim Behav 38: 486–495 (doi: 10.1016/S0003-3472(89)80041-7).

[5] Menzel R, Greggers U, Smith A, Berger S, Brandt R, et al (2005) Honey bees navigate according to a map-like spatial memory. Proc Natl Acad Sci USA 102: 3040–3045 (doi: 10.1073/pnas.0408550102). 15710880 10.1073/pnas.0408550102 PMC549458

[6] Tarsitano M, Andrew R (1999) Scanning and route selection in the jumping spider *Portia labiata* . Anim Behav 58: 255–265 (doi: 10.1006/anbe.1999.1138). 10458876 10.1006/anbe.1999.1138

[7] Brown C, Laland KN, Krause J (2011) Fish Cognition and Behavior, 2nd edition. Cambridge: Blackwell Publishing.

[8] Grosenick L, Clement TS, Fernald RD (2007) Fish can infer social rank by observation alone. Nature 445: 429–432 (doi:10.1038/nature05511). 17251980 10.1038/nature05511

[9] Kendal JR, Rendall L, Pike TW, Laland KN (2009) Nine-spined sticklebacks deploy a hill-climbing social learning strategy. Behav Ecol 20: 238–244 (doi: 10.1093/beheco/arp016).

[10] Randall JE (1958) A review of the labrid fish genus Labroides, with descriptions to two new species and notes on ecology. Pac Sci 12: 327–347.

[11] Grutter AS, Bshary R (2003) Cleaner wrasse prefer client mucus: support for partner control mechanisms in cleaning interactions. Proc R Soc Lond B 270: 242–244 (doi:10.1098/rsbl.2003.0077). 10.1098/rsbl.2003.0077 PMC1809949 14667394

[12] Bshary R (2010) Cooperation between unrelated individuals - a game theoretic approach. In: Kappeler PM, editor. Animal Behavior: Evolution and Mechanisms. Berlin: Springer. pp. 213–240.

[13] Bshary R (2011) Machiavellian intelligence in fishes. In: Brown C, Laland KN, Krause, J., editors. Learning and Cognition in Fishes, 2nd edition. Oxford: Blackwell. pp. 277–279.

[14] Pinto AI, Oates J, Grutter AS, Bshary R (2011) Cleaner wrasses *Labroides dimidiatus* are more cooperative in the presence of an audience. Curr Biol 21: 1140–1144 (doi: 472 10.1016/j.cub.2011.05.021). 21700458 10.1016/j.cub.2011.05.021

[15] Grutter AS (1995) Relationship between cleaning rates and ectoparasite loads in coral reef fishes. Mar Ecol Prog Ser 118: 51–58.

[16] Bshary R, Grutter AS (2002) Experimental evidence that partner choice is a driving force in the payoff distribution among cooperators or mutualists: the cleaner fish case. Ecol Lett 5: 130–136 (doi: 10.1046/j.1461-0248.2002.00295.x).

[17] Barbu L, Guinand C, Bergmüller R, Alvarez N, Bshary R (2011) Cleaning wrasse species differ with respect to client reef fish composition and behavioural adaptations to cleaning interactions. Anim Behav 82: 1067–1074 (doi: 10.1016/j.anbehav.2011.07.043).

[18] Bshary R (2001) The cleaner fish market. In Economics in Nature (ed. R. Noë, J. A. R. A. M. 483 Van Hooff & P. Hammerstein), pp. 146–172. Cambridge: Cambridge University Press.

[19] Deaner RO, Van Schaik CP, Johnson V (2006) Do some taxa have better domain general cognition than others? A meta-analysis of nonhuman primate studies. Ev Psychol 4: 149–196.

[20] Emery NJ (2006) Cognitive ornithology: the evolution of avian intelligence. Phil Trans R Soc Lond B 361: 23–43 (doi: 10.1098/rstb.2005.1736). 16553307 10.1098/rstb.2005.1736 PMC1626540

[21] Reader SM, Hager Y, Laland KN (2011) The evolution of primate general and cultural intelligence. Phil Trans R Soc Lond B 366: 1017–1027 (doi: 10.1098/rstb.2010.0342). 21357224 10.1098/rstb.2010.0342 PMC3049098

[22] Shultz S, Dunbar RIM (2010) Species differences in executive function correlate with hippocampus volume and neocortex ratio across nonhuman primates. J Comp Psychol 124: 252–260 (doi: 10.1037/a0018894). 20695656 10.1037/a0018894

[23] Boesch C (1994) Cooperative hunting in wild chimpanzees. Anim Behav 48: 653–667 (doi: 10.1006/anbe.1994.1285,).

[24] Fragaszy DM, Visalberghi E, Fedigan LM (2004) The Complete Capuchin: The biology of the genus Cebus. Cambridge: Cambridge University Press.

[25] Rose LM (1997) Vertebrate predation and food-sharing in Cebus and Pan. Int J Primatol 18: 727–765 (doi: 10.1023/A:1026343812980).

[26] Brosnan SF (2010) What do capuchin monkeys tell us about cooperation? In: Fosyth DR, Hoyt CL, editors. For the Greater Good of All: Perspectives on Individualism, Society, and Leadership Perspectives on Individualism, Society, and Leadership, vol. Jepson Studies in Leadership Series. NYC: Palgrave Macmillan Publishers. pp. 11–28.

[27] Brosnan SF, Freeman C, de Waal FBM (2006) Partner's behavior, not reward distribution, determines success in an unequal cooperative task in capuchin monkeys. Am J Primatol 68: 713–724 (doi: 10.1002/ajp.20261). 16786518 10.1002/ajp.20261

[28] Chalmeau R, Lardeux K, Brandibas P, Gallo A (1997) Cooperative problem solving by orangutans (*Pongo pygmaeus*). Int J Primatol 18: 23–32 (doi: 509 10.1023/A:1026337006136).

[29] Dufour V, Pele M, Neumann M, Thierry B, Call J (2009) Calculated reciprocity after all: computation behind token transfers in orang-utans. Biol Lett 5: 172–175 (doi: 512 10.1098/rsbl.2008.0644). 19126529 10.1098/rsbl.2008.0644 PMC2665816

[30] Melis AP, Hare B, Tomasello M (2006) Chimpanzees recruit the best collaborators. Science 311: 1297–1300 (doi: 10.1126/science.1123007). 16513985 10.1126/science.1123007

[31] Melis AP, Hare B, Tomasello M (2006) Engineering cooperation in chimpanzees: Tolerance constraints on cooperation. Anim Behav 72: 275–286 (doi: 517 10.1016/j.anbehav.2005.09.018).

[32] Mendres KA, de Waal FBM (2000) Capuchins do cooperate: the advantage of an intuitive task. Anim Behav 60: 523–529 (doi: 10.1006/anbe.2000.1512). 11032655 10.1006/anbe.2000.1512

[33] Perry S, Manson JH, Dower G, Wikbert E (2003) White-faced capuchins cooperate to rescue a groupmate from a Boa constrictor. Folia Primatol 74: 109–111 (doi: 522 10.1159/000070008). 12778926 10.1159/000070008

[34] Dufour V, Pele M, Sterck EHM, Thierry B (2007) Chimpanzee (*Pan troglodytes*) anticipation of food return: Coping with waiting time in an exchange task. J Comp Psychol 121: 145–155 (doi: 10.1037/0735-7036.121.2.145). 17516793 10.1037/0735-7036.121.2.145

[35] Pelé M, Dufour V, Micheletta J, Thierry B (2010) Long-tailed macaques display unexpected waiting abilities in exchange tasks. Anim Cogn 13: 263–271 (doi: 528 10.1007/s10071-009-0264-6). 19597853 10.1007/s10071-009-0264-6

[36] Döhl J (1968) Über die Fähigkeit einer Schimpansin, Umwege mit selbständigen Zwischenzielen zu überblicken. Z Tierpsychol 25: 89–103 (doi: 10.1111/j.1439531 0310.1968.tb00005.x). 5698407

[37] Mulcahy N, Call J (2006) Apes save tools for future use. Science 312: 1038–1040 (doi: 533 10.1126/science.1125456). 16709782 10.1126/science.1125456

[38] Osvath M (2009) Spontaneous planning for future stone throwing by a male chimpanzee. Curr Biol 19: R190–R191 (doi: 10.1016/j.cub.2009.01.010). 19278627 10.1016/j.cub.2009.01.010

[39] Osvath M (2010) Great ape foresight is looking great. Anim Cogn 13: 777–781 (doi: 10.1007/s10071-010-0336-7). 20607575 10.1007/s10071-010-0336-7

[40] Deaner RO, Isler K, Burkart J, van Schaik CP (2007) Overall brain size, and not encephalization quotient, best predicts cognitive ability across non-human primates. Brain Behav Evol 70: 115–124 (doi: 10.1159/000102973). 17510549 10.1159/000102973

[41] Rumbaugh DM (1971) Evidence of qualitative differences in learning processes among primates. J Comp Physiol Psychol 76: 250–255 (doi: 10.1037/h0031401). 5003545 10.1037/h0031401

[42] Rumbaugh DM, Pate JL (1984) The evolution of cognition in primates: A comparative perspective. In: Roitblat HL, Bever TG, Terrace HS, eds. Animal Cognition. Hillsdale, NJ: Erlbaum. pp. 569–587.

[43] Chikka L, Jensen K (2011) Animal cognition: Concepts from apes to bees. Curr Biol 21: R116–R119 (doi; 10.1016/j.cub.2010.12.045). 21300275 10.1016/j.cub.2010.12.045

[44] Beran MJ (2001) Summation and numerousness judgments of sequentially presented sets of items by chimpanzees (Pan troglodytes). J Comp Psychol 155: 181–191 (10.1037/0735-7036.115.2.181). 10.1037/0735-7036.115.2.181 11459165

[45] Beran MJ, Evans TA, Leighty KA, Harris EH, Rice D (2008) Summation and quantity judgments of sequentially presented sets by capuchin monkeys (*Cebus apella*). Am J Primatol 70: 191–194 (doi: 10.1002/ajp.20474). 17879377 10.1002/ajp.20474

[46] Evans TA, Beran MJ, Harris EH, Rice D (2009) Quantity judgments of sequentially presented food items by capuchin monkeys (*Cebus apella*). Anim Cogn 12: 97–105 (doi: 558 10.1007/s10071-008-0174-z). 18670794 10.1007/s10071-008-0174-z

[47] Hanus D, Call J (2007) Discrete quantity judgments in the great apes (*Pan paniscus, Pan troglodytes, Gorilla gorilla, Pongo pygmaeus*): The effect of presenting whole sets versus item-by-item. J Comp Psychol 121: 241–249 (doi: 10.1037/0735-7036.121.3.241). 17696650 10.1037/0735-7036.121.3.241

[48] Agrillo C, Dadda M, Bisazza A (2007) Quantity discrimination in female mosquitofish. Anim Cogn 10: 63–70 (doi: 10.1007/s10071-006-0036-5). 16868736 10.1007/s10071-006-0036-5

[49] Agrillo C, Dadda M, Serena G, Bisazza A (2008) Do fish count? Spontaneous discrimination of quantity in female mosquitofish. Anim Cogn 11: 495–503. 18247068 10.1007/s10071-008-0140-9

[50] Addessi E, Crescimbene L, Visalberghi E (2008) Food and quantity token discrimination in capuchin monkeys (*Cebus apella*). Anim Cogn 11: 275–282 (10.1007/s10071-008568 0140-9). 17901990 10.1007/s10071-007-0111-6

[51] Beran MJ, Harris EH, Evans TA, Klein ED, Chan B, et al (2008) Ordinal judgments of symbolic stimuli by capuchin monkeys (*Cebus apella*), and rhesus monkeys (*Macaca mulatta*): The effects of differential and nondifferential reward. J Comp Psychol 122: 52–61 (doi: 10.1037/0735-7036.122.1.52). 18298281 10.1037/0735-7036.122.1.52

[52] Evans TA, Beran MJ, Addessi E (2010) Can nonhuman primates use tokens to represent and sum quantities? J Comp Psychol 124: 369–380 (doi: 10.1037/a0019855). 20836596 10.1037/a0019855 PMC5152591

[53] Bshary R, Würth M (2001) Cleaner fish *Labroides dimidiatus* manipulate client reef fish by providing tactile stimulation. Proc R Soc Lond B 268: 1495–1501. 10.1098/rspb.2001.1495 PMC1088769 11454294

[54] Dickinson A, Balleine B (1994) Motivational control of goal directed action. Anim Learn Behav 22: 1–18 (doi 10.3758/BF03199951).

[55] Deaner RO, Isler K, Burkart J, van Schaik C (2007) Overall Brain Size, and Not Encephalization Quotient, Best Predicts Cognitive Ability across Non-Human Primates. Brain Behav Evol 70: 115–124 (doi: 10.1159/000102973). 17510549 10.1159/000102973

[56] Deaner RO, van Schaik CP, Johnson V (2006) Do some taxa have better domain-general cognition than others? A meta-analysis of nonhuman primate studies. Ev Psychol 4: 149–196.

[57] Oates J, Manica A, Bshary R (2010a) Roving decreases service quality in the cleaner wrasse *Labroides bicolor* . Ethology 116: 309–315.

[58] Oates J, Manica A, Bshary R (2010b) The Shadow of the Future affects cooperation in cleaner fish. Curr Biol 20: R472–R473. 20541490 10.1016/j.cub.2010.04.022

[59] Cruze WW (1935) Maturation and learning in chicks. J Comp Psychol 19: 371–409 (doi: 578 10.1037/h0063532).

[60] Bisazza AL, Piffer SG, Agrillo C (2010) “Ontogeny of numerical abilities in fish.”. PLoS ONE 5: e15516. 21124802 10.1371/journal.pone.0015516 PMC2991364

[61] Brown C, Laland KN, Krause J, eds (2011) *Learning and Cognition in Fishes*. Oxford: Blackwell

[62] Kendal JR, Rendell L, Pike TW, Laland KN (2009) Nine-spined sticklebacks deploy a hill-climbing social learning strategy. Behav Ecol 20: 238–244.

[63] Pike TW, Laland KN (2010) Conformist learning in nine-spined sticklebacks' foraging decisions. Biol Lett 6: 466–468. 20129948 10.1098/rsbl.2009.1014 PMC2936200

[64] Grosenick L, Clement TS, Fernald RD (2007) Fish can infer social rank by observation alone. Nature 445: 429–432. 17251980 10.1038/nature05511

[65] Helfman GS, Schultz ET (1984) Social transmission of behavioural traditions in a coral reef fish. Anim Behav 32: 379–384.

[66] Warner RR (1988) Traditionality of mating site preferences in a coral reef fish. Nature 335: 719–721.

[67] Ferrari MCO, Gonzalo A, Messier F, Chivers DP (2007) Generalization of learned predator recognition: an experimental test and framework for future studies. Proc R Soc Lond B 274: 1853–1859. 10.1098/rspb.2007.0297 PMC2270927 17519190

[68] Bshary R, Hohner A, Ait-el-Djoudi K, Fricke H (2006) Interspecific communicative and coordinated hunting between groupers and giant moray eels in the Red Sea. PLoS Biol 4: e431. 17147471 10.1371/journal.pbio.0040431 PMC1750927

[69] Agrillo C, Piffer L, Bisazza A, Butterworth B (2012) Evidence for Two Numerical Systems That Are Similar in Humans and Guppies. PLoS ONE 7: e31923 (doi:10.1371/journal.pone.0031923). 22355405 10.1371/journal.pone.0031923 PMC3280231

[70] Agrillo C, Dadda M, Serena G, Bisazza A (2009) Use of Number by Fish. PLoS ONE 4: e4786 (doi:10.1371/journal.pone.0004786). 19274079 10.1371/journal.pone.0004786 PMC2650784

[71] Soares MC, Oliveira RF, Ros AFH, Grutter AS, Bshary R (2011) Tactile stimulation lowers stress in fish. Nat Commun 2: 1–5. 10.1038/ncomms1547 22086335

[72] Bshary R, Grutter AS, Willener AST, Leimar O (2008) Pairs of cooperating cleaner fish provide better service quality than singletons. Nature 455: 964–967. 18923522 10.1038/nature07184

[73] Bshary R, Wickler W, Fricke H (2002) Fish cognition: a primate's eye view. Anim Cogn 5: 1–13 (doi: 10.1007/s10071-001-0116-5). 11957395 10.1007/s10071-001-0116-5

[74] Salwiczek LH, Bshary R (2011) Cleaner wrasses keep track of the ‘when’ and ‘what’ in a foraging task. Ethology 117: 939–948.

[75] Slocombe KE, Zuberbühler K (2007) Chimpanzees modify recruitment screams as a function of audience composition. Proc Natl Acad Sci USA 104: 17228–17233 (doi: 585 10.1073/pnas.0706741104). 17942683 10.1073/pnas.0706741104 PMC2040427

[76] Byrne RW, Whiten A (1990) Tactical deception in primates: the 1990 database. Primate Report, Whole Volume 27: 1–101.

