# Supplementary material for: Adult Cleaner Wrasse Outperform Capuchin Monkeys, Chimpanzees and Orang-utans in a Complex Foraging Task Derived from Cleaner – Client Reef Fish Cooperation
Source: PLoS One. 2012 Nov 21;7(11):e49068. doi: 10.1371/journal.pone.0049068 (PMC3504063; doi:10.1371/journal.pone.0049068)
Supplement: Information S1 — (DOC) [file pone.0049068.s001.doc]

**Supplemental Methods**

Salwiczek et al

**Cleaner Fish**

Cleaner fish were housed in 25 l of saltwater with a salinity between 1.020 – 1.026 as measured with a MR 100 ATC refractometer (Milwaukee MR series refractometer). Nitrite was measured every second day with a TETRA test set (Mat.-No. 326 972 CE – T613, Tetrawerke, D-49304 Melle) and water was changed if the nitrite concentration reached 0.8 mg/l. Every aquarium was equipped with an Eheim aquaball 2208 modular internal filter (for technical specification see Table S1); and a dark grey opaque Plexiglas tube (length: 10cm, Ø = 2.5 cm) for hiding during the day and sleeping at night. A sliding door was introduced at two-thirds of the aquarium lengths: The right and left wing of the grey opaque Plexiglas door (width: 11cm, height: 29cm) remained in the aquarium throughout the experiments, while the middle mobile part (width: 7cm, height: 29cm) was removed outside experiments. The gap between the two door-wings was 6.5 cm. The fish were kept on a 12:12 hr day: night schedule. Room temperature was 25ºC and water temperature 25ºC."

Table S1: Eheim aquaball 2208, Eheim 73555 90 MSS/04.02

| liter | 30 - 60 |
| --- | --- |
| l/h | 480 |
| Hmax m | 0.37 |
| W | 5 |
| cm^3^ | 185 |
| mmL | 160 |
| mm Ø | 96 |
| V | 230 |
| Hz | 50 |

**Capuchin Monkeys**

Capuchin monkeys were housed in two groups at the Language Research Center of Georgia State University in Atlanta, Georgia, USA. One group consisted of 2 adult males, 2 adult females, and 2 juveniles. The other consisted of 1 adult male, 2 sub-adult males, 2 adult females, and 1 juvenile. Age for these subjects ranged from 3 to 21 years for males (average 9.57 ± SD 6.68 years, N = 7), to 3 to 14 years for females (average 9.8 ± SD 4.55 years, N = 5). For both groups, enclosures consisted of an indoor room (Group 1: 25.28m^2^, Group 2: 18.14 m^2^) connected to a large outdoor enclosure (Group 1: 13.51m^2^ ; Group 2: 21.15m^2^). Group members spent most of their time in the outdoor area throughout the year, except when engaged in research, during bad weather, or overnight. The indoor-outdoor enclosures were made of chain link fencing, including the ceiling, and were equipped with swings, ropes, and other materials to create three-dimensional living conditions to enrich the monkeys. All subjects participated in testing voluntarily, being called in from their social groups and tested in testing boxes attached to their living area. All monkeys had *ad libitum* access to water and were fed chow, fresh fruits and vegetables, and enrichment items several times throughout the day. Animals were never food or water deprived for the purposes of testing. The Institutional Animal Care and Use Committee of Georgia State University approved all testing procedures.

**Chimpanzees**

We tested four chimpanzees housed at the Language Research Center of Georgia State University.  Subjects consisted of two adult males (ages 36 and 23) and two adult females (ages 39 and 24), all of whom lived in a cognitively enriched environment and had undergone extensive cognitive testing since infancy. All four chimpanzees were highly experienced in a variety of different cognitive tests, including exchange tests (Brosnan et al., 2008), delay of gratification tests (Beran & Evans, 2006), and tests in which they made judgments of food quantities (Beran & Beran, 2004).  All chimpanzees lived together in a social group in a building that allowed both indoor and outdoor access, including vertical space and other enrichment. Subjects were separated only for daily test sessions into individual test areas which were part of their living space.  All subjects participated voluntarily, being called in from their social groups and tested in one of the indoor dens of their living area. All chimpanzees had *ad libitum* access to water and were fed chow, fresh fruits and vegetables, and enrichment items several times throughout the day. Animals were never food or water deprived for the purposes of testing. The Institutional Animal Care and Use Committee of Georgia State University approved all testing procedures.

**Orangutans**

Orangutans were housed in social groups at Zoo Atlanta, Atlanta, GA, USA. All subjects had indoor/outdoor access and extensive material enrichment (climbing structures, ropes and swings, barrels, and other toys). Tests subjects came from two social groups. Group 1 consisted of a 34 year old hybrid Sumatran/Bornean male, 28 year old Sumatran female, and two Sumatran juvenile males (ages 9 and 5), and an infant (1 year old) Sumatran male. Group 2 consisted of all Bornean orangutans, including an adult male (age 18), a subadult male (age 8) and an adult female (age 19). The Sumatran female test subject was reared in a computer-enriched environment at the Georgia State University Language Research Center (Washburn et al., 2007) and participated in cognitive tasks there (Beran, 2002). The adult male had previous experience using sign language to communicate with humans. She and the two subadult males had previous training with a variety of cognitive tasks using the matching-to-sample paradigm on a computerized-touchscreen testing apparatus including matching social stimuli such as faces.

All subjects were fed their usual diet consisting of primate chow, fruits and vegetables throughout the course of the study. In addition, feeding enrichment was provided on a daily basis as part of the husbandry routine. At no time were the subjects ever food or water deprived. Studies involved a single ape at a time. All subjects participated voluntarily, being called in from their social groups and tested in one of the indoor dens of their living area. If possible subjects were separated from other individuals to limit distractions (unweaned infants always accompanied their mothers). All procedures used in this research were approved by the Scientific Review Committee of Zoo Atlanta and the Institutional Animal Care and Use Committee of Georgia State University.

**References**

Beran MJ (2002) Maintenance of self-imposed delay of gratification by four chimpanzees (*pan troglodytes*) and an orangutan (*Pongo pygmaeus*). J Gen Psychol 129:49-66.

Beran MJ, Beran MM (2004) Chimpanzees remember the results of one-by-one addition of food items to sets over extended time periods. Psychol Sci 15: 94-99.

Beran MJ, Evans TA (2006) Maintenance of delay of gratification by four chimpanzees (*Pan troglodytes*): The effects of delayed reward visibility, experimenter presence, and extended delay intervals. Behav Process 73: 315-324.

Brosnan SF, Grady M, Lambeth S, Schapiro S, Beran MJ (2008) Chimpanzee autarky. PLoS ONE 3: e1518.

Washburn DA, Gulledge JP, James F, Rumbaugh DM (2007) A species difference in visuospatial working memory: Does language link "What" With "Where"? Int J Comp Psychol 20: 55-64.
